# Laccase *versus* Laccase-Like Multi-Copper Oxidase: A Comparative Study of Similar Enzymes with Diverse Substrate Spectra

**DOI:** 10.1371/journal.pone.0065633

**Published:** 2013-06-03

**Authors:** Renate Reiss, Julian Ihssen, Michael Richter, Eric Eichhorn, Boris Schilling, Linda Thöny-Meyer

**Affiliations:** 1 Laboratory for Biomaterials, Empa, Swiss Federal Laboratories for Materials Science and Technology, St. Gallen, Switzerland; 2 Givaudan Schweiz AG, Dübendorf, Switzerland; Instituto de Tecnologica Química e Biológica, UNL, Portugal

## Abstract

Laccases (EC 1.10.3.2) are multi-copper oxidases that catalyse the one-electron oxidation of a broad range of compounds including substituted phenols, arylamines and aromatic thiols to the corresponding radicals. Owing to their broad substrate range, copper-containing laccases are versatile biocatalysts, capable of oxidizing numerous natural and non-natural industry-relevant compounds, with water as the sole by-product. In the present study, 10 of the 11 multi-copper oxidases, hitherto considered to be laccases, from fungi, plant and bacterial origin were compared. A substrate screen of 91 natural and non-natural compounds was recorded and revealed a fairly broad but distinctive substrate spectrum amongst the enzymes. Even though the enzymes share conserved active site residues we found that the substrate ranges of the individual enzymes varied considerably. The EC classification is based on the type of chemical reaction performed and the actual name of the enzyme often refers to the physiological substrate. However, for the enzymes studied in this work such classification is not feasible, even more so as their prime substrates or natural functions are mainly unknown. The classification of multi-copper oxidases assigned as laccases remains a challenge. For the sake of simplicity we propose to introduce the term “laccase-like multi-copper oxidase” (LMCO) in addition to the term laccase that we use exclusively for the enzyme originally identified from the sap of the lacquer tree *Rhus vernicifera*.

## Introduction

Multi-copper oxidases (MCOs) belong to a protein superfamily of enzymes which oxidize the substrate at a mononuclear copper center T1. Electrons are then transferred internally to the trinuclear copper center T2/T3 where dioxygen is reduced by four electrons, yielding two water molecules. The copper ions in the active sites are classified as type-1 (T1), type-2 (T2) and type-3 (T3) according to their spectroscopic properties [1]. Thus, at least four copper atoms are present in MCOs and shared between the T1 and T2/T3 sites [2].

Although the sequence homology among MCOs is low, amino acid alignments show that the overall structures and copper-binding motifs are highly conserved [3]. The T1 copper atom is bound by two histidine residues and one cysteine residue, which forms a metallo-organic bond. In addition, the side chains of a methionine, a leucine or an isoleucine are in close proximity to the T1 copper [4]. The T2 copper atom of the trinuclear center is coordinated by two histidine ligands, while the two T3 coppers have in total six histidine ligands. The copper ligands are organized in four subsequent, strictly conserved motifs within the primary amino acid sequence HXHG, HXH, HXXHXH and HCHXXXHXXXXM/L/F. From the structural point of view, MCOs comprise 2, 3 or 6 homologous domains, each resembling a cupredoxin-like fold arranged in an (eight-stranded) Greek-key beta-barrel consisting of two β-sheets, each containing four strands [3], [5].

Laccases (EC 1.10.3.2), ascorbate oxidase (EC 1.10.3.3), ferroxidase (EC 1.16.3.1) nitrite reductase (EC 1.7.2.1) and ceruloplasmin (EC 1.16.3.1) are all members of this multi-copper oxidase superfamily.

In a typical reaction cycle, the largest subgroup of MCOs, the so-called laccases catalyze the iterative one electron oxidation of four substrate molecules including polyphenols, polyamines and certain inorganic compounds with the simultaneous reduction of molecular oxygen to water. Syringaldazine was suggested to be a substrate unique to laccases in the absence of hydrogen peroxide [6], [7], [8]. Overlapping oxidation activity was found with tyrosinase, pholyphenol oxidase and bilirubin oxidase, which hampers the classification [6], [7], [9], [10]. For example, we identified, characterized and classified a new CotA-type enzyme from *Bacillus pumilus* as laccase [11]. Recently, Durad et al. also studied this *B. pumilus* enzyme with 98% sequence identity to our laccase and assigned it as bilirubin oxidase [12]. This activity has also been reported for a *Bacillus subtilis* CotA-type laccase [9]. Hence, a narrow definition of this MCO subgroup by substrates seems inadequate as these enzymes are active on a broad range of natural and synthetic compounds.

Attempts have been made to classify plant and fungal as well as bacterial laccases based on sequence analysis [13], [14], [15]. Laccase specific amino acid signature sequences other than the histidine motifs were deduced from plant and fungal sequences. However, the functional significance of these signature sequences as well as whether they are applicable for a meaningful analysis of bacterial MCO sequences are unclear. In general, the discrimination of laccases from other MCOs is challenging and requires more experimental data. Most sequences in genome databases annotated as laccases still await experimental verification. So far, there is no unifying amino acid pattern described for laccase identification in a distinctive and concise way.

To date, it cannot be defined exactly what a true laccase is. MCOs have been assigned as laccases provided that the four copper atoms are present in the typical formation of type-1, type-2 and type-3 copper centers and that some phenol oxidase activity can be measured [16], [17]. The definition is further complicated because laccases are above all a diverse subgroup of MCOs, spanning from plant to fungal to bacterial taxonomic genera [16]. Clearly, there is a strong need for accurate classification. It has also been suggested, that only MCOs isolated from plant saps in the presence of the natural substrate urushiol, an unsaturated alkylcatechol, should be named laccases, referring to the first identified “type” laccase. We also propose to introduce the term laccase-like MCOs (LMCOs) in order to account for the potential multiplicity of their biological functions [16], [17]. For the sake of simplicity all studied enzymes will be referred to as LMCO in this work, with the exception of the *Rhus vernicifera* laccase (the “type laccase”) and the *Cucurbita* ascorbate oxidase.

Laccases were discovered in the sap of the japanese lacquer tree *R. vernicifera*, but LMCOs have been found in numerous other plants, insects, bacteria and fungi [18]. The biological functions of laccases and LMCOs are diverse and presumably include the formation and degradation of lignin in the case of plant and fungal enzymes, the biosynthesis of a brown spore pigment by CotA from *B. subtilis* and the production of external cuticle in insects [19]. In most cases neither the natural reaction(s) performed by LMCOs nor their substrate(s) are known. LMCOs have gained considerable attention in recent years in biotechnology due to several intrinsic advantages compared to other oxidative enzymes: broad substrate range, high stability, simple application (no need for external supply, recycling of cofactors and water as sole byproduct). LMCOs have been shown to bear high potential for alternative, green chemistry synthesis routes as well as for diverse applications in the textile, paper and wood industries [20]. The presently known laccases and LMCOs are particularly difficult to overproduce in heterologous hosts [21]. Therefore, there is a need for novel LMCOs with potentially higher yields in industrial production organisms.

In the present study, 11 laccases and LMCOs of bacterial, fungal and plant origin were compared by focusing on two technically important characteristics, the pH range and the substrate spectrum. A UV-Vis spectrum based screen comprising 91 potential substrates was performed.

The purpose of this study was to provide a comparative characterization of laccases and LMCOs from various sources in an effort to contribute to a more comprehensive understanding of this biotechnologically important class of enzymes.

## Materials and Methods

### Materials

2,2′-Azino-bis(3-ethylbenzothiazoline-6-sulphonic acid) (ABTS), 4-hydroxy-3,5-dimethoxybenzaldehyde azine (syringaldazine, SGZ), 2,6-dimethoxyphenol (2,6-DMP), 3′, 5′-dimethoxy-4′-hydroxyacetophenone (acetosyringone) (ACS) and all compounds used for the substrate screen were purchased from Sigma-Aldrich (standard reagent grade). Freeze-dried preparations of *Rhus vernificera* laccase (p-Rve), *Trametes versicolor* LMCO (f-Tve) and *Cucurbita* ascorbate oxidase (p-Cur) were purchased from Sigma-Aldrich. *Agaricus bisporus* LCMO was purchased from ASA Spezialenzyme GmbH and *Myceliophthora thermophila* LMCO (f-Mth) was purchased from Novozymes.

### Recombinant bacterial MCOs

LCMOs of bacterial origin were expressed in recombinant form in *Escherichia coli*. Genome mining, cloning and expression studies for the plasmids used in this study are described elsewhere. Here we used the best-performing plasmids and expression conditions [manuscript in preparation]. *E. coli* BL21(DE3) (pBuL) was used for expression of *B. pumilus* DSM27 CotA LMCO, *E. coli* BL21(DE3) (pLOM10) was used for expression of *B. subtilis* CotA LMCO [22]. *E. coli* BL21(DE3) (pGoL3) was used for expression of *Gramella forsetii* KT0803 LMCO, *E.coli* JM109 (pSpL3) was used for expression of *Streptomyces pristinaespiralis* DSM40338 LMCO, *E. coli* BL21(DE3) (pMtraL) used for expression of *Marivirga tractuosa* DSM4126 LMCO and *E. coli* BL21(DE3) (pSiL) was used for expression of *Spirosoma linguale* DSM74 LCMO [manuscript in preparation].For expression of recombinant bacterial MCOs either LB (5 g/L yeast extract, 10 g/L tryptone, 5 g/L NaCl) or LBPG (5 g/L yeast extract, 10 g/L tryptone, 7.25 g/L Na_2_HPO_4_ • 2H_2_O, 3 g/L KH_2_PO_4_, 4 g/L glucose) medium was used. All media were supplemented with 100 mg/L ampicillin for plasmid selection. Shake flask cultures (total volume 200–300 mL, liquid volume 60 mL) were inoculated 1∶50 from fresh overnight tube precultures (37°C, 160 rpm) and incubated at 30°C and 160 rpm until an OD_600_ of 0.4–0.5 was reached. Subsequently, the cultivation temperature was reduced to 25°C. The expression of recombinant proteins was induced by the addition of IPTG to a final concentration of 1 mM, and CuCl_2_ was added to a final concentration of 0.25 mM. LB cultures were subsequently incubated for 4 h at 100 rpm and then shifted to static incubation for overnight expression. LBPG cultures were shifted to static incubation at 25°C directly after induction. Static incubation leading to oxygen-limited growth was shown previously to increase the yield of fully copper loaded bacterial LMCO in *E. coli* [23].

Cells were harvested after overnight incubation by centrifugation for 1 min at 12000× g, and cell pellets were frozen at −80°C. Pellets were re-thawed to a calculated OD_600_ of 10 in 1× CellLytic B solution (Sigma-Aldrich) containing 0.2 mg/mL lysozyme and 0.5 µL/mL Benzonase® Nuclease (Novagen). Lysis reactions were incubated for 20 min at room temperature and 200 rpm, and cell debris was removed by centrifugation (5 min, 12000× g). Supernatants (cell free extract, CFE) were used for pH dependency studies.

CFE containing recombinant bacterial LMCOs and CFE of an empty vector control strain (*E. coli* BL21(DE3) transformed with pET-22b(+)) for the substrate determination assay were prepared using LB medium as described previously [11]. Briefly, cells were grown with a static induction phase, lysed by sonication in the presence of lysozyme, Benzonase® Nuclease and protease inhibitor, and the supernatant was recovered by centrifugation and used without further dilution in the assay.

### Biochemical assays

Enzymatic activity was determined at room temperature. The assay solution contained 5 mM ABTS as substrate in 50 mM tartaric acid buffer pH 4, and transparent polystyrene 96-well microplates (Nunc) were used. The reaction was followed with a microplate reader at 420 nm, and enzymatic activity was calculated with a molar extinction coefficient for oxidized ABTS of 36'000 M^−1^ cm^−1^.

The pH optimum and range of bacterial and eukaryotic LMCOs was determined in triplicate using the McIlvaine citrate buffer row series in the pH range 2.2–8.0. In the range of pH 8.5–9.5 100 mM Tris-hydroxyaminomethane-HCl was used as buffer, while in the range of pH 10.0–12.0 200 mM sodium phosphate buffer was used. The substrates ABTS and 2,6-DMP each were added to a final concentration of 5 mM, and the assays were performed in parallel in 96-well microtiter plates at room temperature. Relative activities for 2,6-DMP could not be reliably determined above pH 8.0 due to autooxidation of the substrate.

The substrate range of bacterial and commercial LMCOs and laccase was determined as single measurement in 96-well plates. Therefore, potential laccase substrates were dissolved in the appropriate solvent at a concentration of 10 mM and diluted to a final concentration of 1 mM in the assay. Routinely, substrates were dissolved in water containing 5% (v/v) DMSO, except for *trans*-cinnamic acid, *p*-coumaric acid, caffeic acid, ferulic acid, syringic acid, methylsyringate, resveratrol, quercetin and phenolphtalein, which were dissolved in ethanol, 4-(dimethylamino) benzoic acid, 3-hydroxyanthranilic acid, 4-amino salicylic acid, *N*-hydroxyphthalimide, epicatechin, 3,5-demethoxy-benzonitrile, which were dissolved in ethanol and methanol 1∶5 (v/v), syringaldazine and phenothiazine, which were dissolved in 100% DMSO and triphenylamine, which was dissolved in THF.

The 200 µL reactions were performed in 96-well plates in 0.1 M potassium phosphate buffer, pH 6.0 at 37°C with shaking at 100 rpm. The reaction was initiated by adding 10 µL CFE or solutions of commercial laccase or LMCO. Control reactions for recombinant bacterial LMCOs contained 10 µL CFE of the empty vector strain. Commercial laccase and LMCO preparations were prepared as stock solutions in ddH_2_O with 100 U/mL of *R. vernificera* laccase, *T. versicolor* LMCO (f-TvL), *M. thermophila* LMCO (f-MtL) and *Cucurbita* ascorbate oxidase (p-Cur). Control reactions contained the appropriate inactivated enzyme, which was previously incubated at 95°C for 10 min. A blank reaction lacking mediator in the co-solvents was also monitored. A UV-Vis scan between 230–700 nm was recorded prior to enzyme addition and after 24 hours reaction time.

## Results and Discussion

### General characteristics and primary structure of the analyzed LMCOs

**Figure 1:**
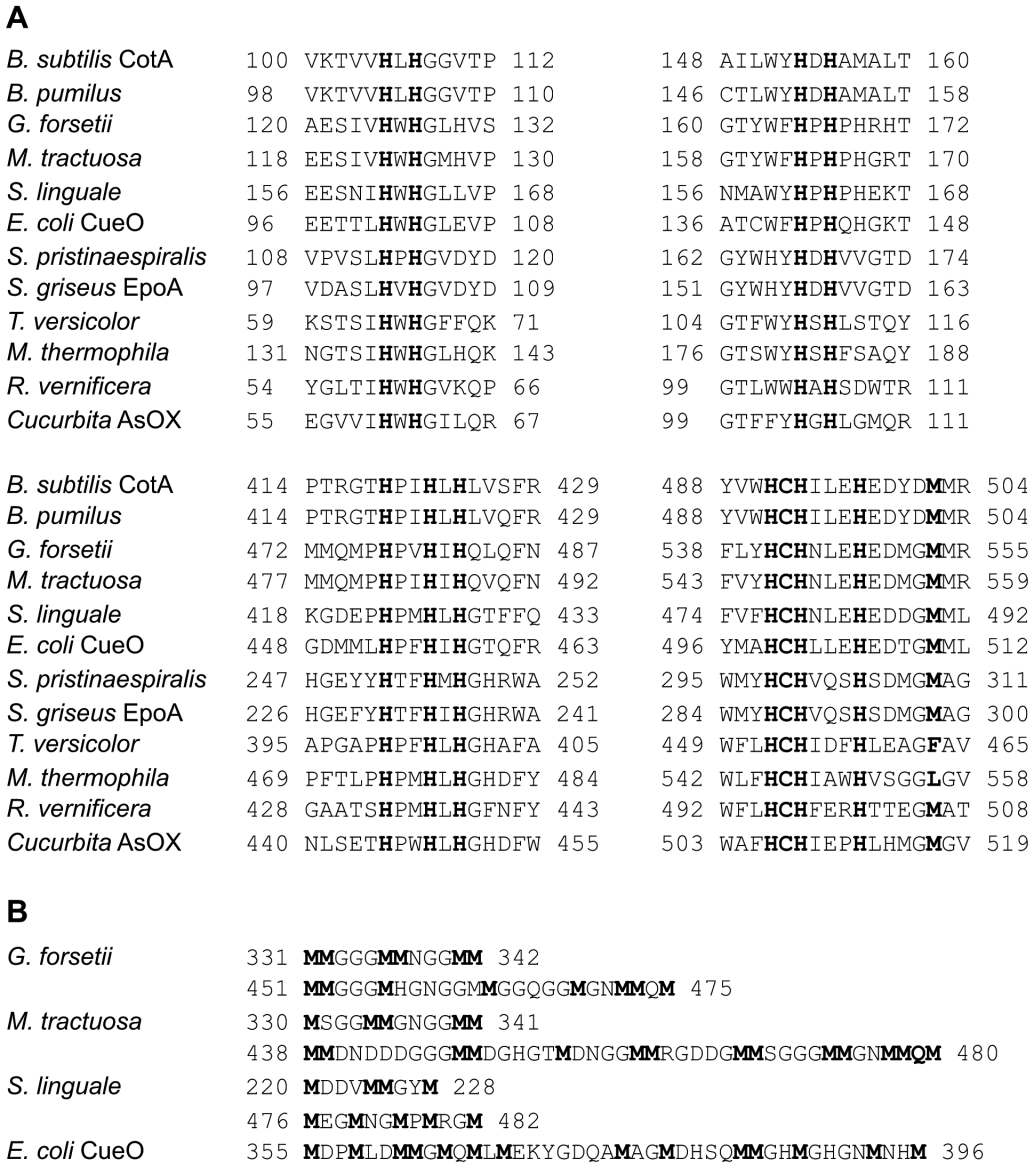
Analysis of the primary structure of the bacterial, fungal and plant LMCOs and laccase used in this study. (A) Alignment in the vicinity of the four copper binding motifs. (B) Methionine-rich regions of LMCOs of Gram-negative bacteria. Sequences of *S. griseus* EpoA and *E. coli* CueO were included for comparison.

In this study we performed a cross-comparison of LCMOs from 11 different organisms, covering diverse genera of Gram-negative and Gram-positive bacteria, fungi and plants, including both secreted and non-secreted enzymes (Table 1). The predicted molecular weight and the amino acid length of the studied enzymes was in a similar range (51–66 kDa, 470–600 aa), with the exception of the *Streptomyces* LMCO (32.6 kDa, 297 aa).The alignment of the primary structures shows that all proteins contain the four strictly conserved copper ligand motifs (Fig. 1A). Significant differences between different phylogenetic groups with respect to residues surrounding the histidine and cysteine copper ligands are rare. LMCOs of Gram-negative bacteria differ from all other sequences at the second histidine motif (HXH) by exhibiting a proline residue between the two histidines and an additional histidine at position two after the motif (Fig. 1A). The *Streptomyces* sequences differ from all other sequences at the third histidine motif (HXXHXH) by lacking any proline in this region, while the other bacterial as well as the fungal and plant sequences exhibit at least one proline residue directly adjacent to the first histidine of the motif (Fig. 1A). In the vicinity of the fourth strictly conserved copper ligand motif (HCHXXXH), the only consistent difference pertains to the plus five position after the last histidine, where only fungal LMCOs have leucine or phenylalanine instead of methionine (Fig. 1A). From the crystal structures of *B. subtilis* and *T. versicolor* LMCOs it is known that the methionine, which is axially coordinated to copper T1 in bacterial LMCOs, is replaced by the non-coordinating residues leucine or phenylalanine [4], [24]. In other regions of the aligned sequences only one distinctive feature was observed which specifically concerned sequences of Gram-negative bacteria. The proteins contained an unusually high number of methionine residues which are clustered in the region in front of the last histidine motif (Fig. 1 B). The region is characterized by repetitive single or double occurrence of methionines with two or three amino acid spacing. In the case of *G. forsetii* and *M. tractuosa* a high number of double or triple glycine residues are found between the methionines. A related motif is present in the LMCO CueO of *E. coli* (Fig. 1 B), and methionine-rich regions have also been reported for CopA of *Stenotrophomonas maltophilia* and McoA of *Aquifex aeolicus*. Different theories have been put forward for assigning a function to these motifs. In the case of the *E. coli* cuprous oxidase CueO it was suggested that the methionines are involved in binding cuprous oxide atoms which are oxidized by the enzyme [25]. For other enzymes it was suggested that copper binding at methionine-rich regions results in a conformational change required for the entry and oxidation of organic substrates [26], [27]. The question of whether these MCOs can be considered LMCOs or constitute a separate class of MCOs remains still open and may be solved by more closely at their substrate spectrum.

**Table 1:** Overview of analyzed laccase and LMCOs.

Organism group	LMCOs analysed in study	Abbr.	Accession No. for amino acid sequence	Amino acid length (mature protein)	MW (mature protein, kDa)	Secreted enzyme	Reference
Gram-positive bacteria	*B. subtilis*	b-Bsu	NP_388511.1	513	58.5	No	[22]
	*B. pumilus*	b-Bpu	ZP_03054403.1	510	58.7	No	[11]
	*S. pristinaespiralis*	b-Spr	ZP_06908025.1	297	32.6	Yes (TAT)	This study
Gram-negative bacteria	*G. forsetii*	b-Gfo	YP_861212.1	526	59.0	Yes (TAT)	This study
	*M. tractuosa*	b-Mtr	YP_004054188.1	532	59.6	Yes (TAT)	This study
	*S. linguale*	b-Sli	YP_003391614.1	470	51.1	Yes (TAT)	This study
Fungi (basidiomycetes)	*T. versicolor*	f-Tve	*PDB: 1KYA	499	53.3	Yes	Sigma-Aldrich
	*A. bisporus*	f-Abi	*AAC18877	520	58	Yes	ASA-Spezial enzyme
Fungi (ascomycetes)	*M. thermophila*	f-Mth	AEO58496.1	595	65.5	Yes	Novozymes
Plants	*R. vernificera*	p-Rve	BAB63411.2	533	59.0	Yes	Sigma-Aldrich
	*Cucurbita* (AsOX)	p-Cur	PDB: 1AOX	552	61.7	Yes	Sigma-Aldrich

Abbreviation: b - bacterial f - fungal, and p - plant.

* Example from the protein database.

### pH profile

The pH optima of laccases and LMCOs are not only dependent on the individual enzyme but also on the type of substrate. Phenolic substrates usually show a bell shape pH profile, while non-phenolic substrates such as ABTS show a monotonic pH profile where rate decreases with increasing pH [28]. In general, the pH optimum for fungal LMCOs is found at basic pH, and for plant and bacterial enzymes at acidic pH. For the majority of fungal LMCOs the pH optimum for hydrogen donating substrates such as 2,6-dimethoxyphenol (DMP) and syringaldazine is in the range of 3.5–5, whereas for electron donor substrates such as ABTS the activity decreases steadily from 2 to 7 [8]. The pH optimum for hydrogen donating substrates for plant and bacterial enzymes laccases and LMCOs such as those from *R. vernicifera* and *Bacillus* species, however, is reported to be in the neutral to weakly alkaline range, while electron donating substrates are oxidized with the highest rates at acidic pH, similarly to what is known from fungal enzymes [11], [18], [22], [29].

To identify the pH at which the studied enzymes perform best in a process, pH dependency studies using two standard laccase substrates, ABTS and DMP, were performed (Table 2). From the results it is evident that fungal LMCOs showed a pH optimum in the acidic range with lower values than for plant and bacterial laccase and LMCOs. This trend was observed for both substrates. The ABTS pH optima were lowest for fungal LMCOs and highest with the phenolic substrate DMP for bacterial LMCOs. The plant laccase from *R. vernificera* can be ranked between the fungal and the bacterial enzymes. Interestingly, the pH range at which the enzymes exhibited activity >50% for ABTS varied considerably. The broadest pH profile was found for LMCO from *B. subtilis*. This is of interest for processes in which enzymes are combined with ABTS or other organic mediators to oxidize a target compound which is not a laccase substrate. These so called laccase mediator systems (LMS) are industrially relevant and are for example used in the textile industry for denim finishing [30], [31]. A specific pH requirement in a process might influence the choice of enzyme. Bacterial LCMOs may be particularly suitable if neutral to basic reaction conditions are required. An additional benefit of bacterial LMCOs is their high stability at elevated temperatures [11]. Data for *S. linguale* MCO was not conclusive due to its low overall activity as shown in Table 3 and was therefore not included in Table 2.

**Table 2:** Relative activity of bacterial and eukaryotic laccase and LMCOs in dependency of the pH with ABTS and 2,6-DMP as substrates.

		ABTS			DMP		
Kingdom	Species	pH range >5% rel. act.	pH range >50% rel. act.	pH opt.	pH range >5% rel. act.	pH range >50% rel. act.	pH opt.
Bacteria	*B. pumilus* (b-Bpu)	2.2–8.1	3.3–5.8	4.7	5.2–≥8.6	6.2–8.1	7.0
	*B. subtilis* (b-Bsu)	2.7–8.6	3.3–7.0	4.2	5.0–≥8.1	6.2–7.8	7.2
	*G. forsetii* (b-Gfo)	3.6–7.0	3.8–4.7	4.2	No activity		
	*M. tractuosa* (b-Mtr)	3.0–6.8	3.8–6.1	4.4	No activity		
	*S. pristinaespiralis* (b-Spr)	3.6–7.5	4.5–5.4	4.7	6.2–≥8.1	7.2–≥8.1	7.6
Fungi	*T. versicolor* (f-Tve)	2.2–5.8	2.2–4.7	2.2	2.2–5.8	2.2–4.4	3.1
	*M. thermophila* (f-Mth)	2.2–5.9	2.2–3.6	2.7	2.2–3.1	2.2–5.9	2.2
Plants	*R. vernificera* (p-Rve)	3.0–6.6	3.4–3.9	3.8	4.4–≥8.1	5.4–≥8.1	5.8

Assays were performed in triplicate at each pH value and the resolution was a pH difference of 0.2. Abbreviation: b - bacterial f - fungal, and p - plant.

**Table 3:** Substrate screen for the 11 studied laccase and LMCOs against the 91 tested substrates.

*No*		Compound	f-Tve	f-Abi	f-Mth	p-Rve	p-Cur	b-Bsu	b-Bpu	b-Spr	b-Gfo	b-Mtr	b-Sli
***1***	**Aromatic carboxylic acids**	*Trans*-cinnamic acid	**−**	**−**	**−**	**−**	**−**	**−**	**−**	**−**	**−**	**−**	**−**
***2***		*m*-Coumaric acid	**+**	**−**	**−**	**−**	**−**	**−**	**−**	**−**	**−**	**−**	**−**
***3***		*p*-Coumaric acid	**+**	**+**	**−**	**−**	**−**	**+/−**	**+/−**	**−**	**−**	**−**	**−**
***4***		Caffeic acid	**+**	**+**	**+**	**+**	**−**	**+**	**+**	**+/−**	**+**	**+**	**+**
***5***		Ferulic acid	**+**	**+**	**+**	**+**	**−**	**+**	**+**	**+/−**	**−**	**+/−**	**+/−**
***6***		Sinapic acid	**+**	**+**	**+**	**+**	**−**	**+**	**+**	**+**	**+/−**	**+**	**+**
***7***		4-Hydroxybenzoic acid	**+**	**−**	**−**	**−**	**−**	**−**	**−**	**−**	**−**	**−**	**−**
***8***		3,4-Dihydroxybenzoic acid	**+**	**+**	**+**	**+**	**−**	**+**	**+**	**−**	**−**	**+/−**	**−**
***9***		Gallic acid	**+**	**+**	**+**	**+**	**−**	**+**	**+**	**−**	**+**	**+**	**−**
***10***		Syringic acid	**+**	**+**	**+**	**+**	**−**	**+**	**+**	**+**	**−**	**+**	**+**
***11***		3-Amino-4-hydroxybenzoic acid	**+**	**+**	**+**	**+**	**−**	**+**	**+**	**−**	**−**	**−**	**−**
***12***		4-Amino-3-hydroxybenzoic acid	**+**	**+**	**+**	**+**	**−**	**+**	**+**	**+**	**+**	**+**	**+**
***13***		3-Fluoro-4-hydroxybenzoic acid	**+**	**−**	**−**	**−**	**−**	**−**	**−**	**−**	**−**	**+/−**	**−**
***14***		4-Dimethylaminobenzoic acid	**+**	**+**	**−**	**−**	**−**	**−**	**−**	**−**	**−**	**+/−**	**−**
***15***		Vanillic acid	**+/−**	**+**	**−**	**−**	**−**	**+/−**	**+/−**	**−**	**−**	**+/−**	**−**
***16***		Anthranilic acid	**+**	**−**	**−**	**−**	**−**	**−**	**−**	**−**	**−**	**+**	**−**
***17***		3-Dimethylaminobenzoic acid	**+**	**−**	**−**	**−**	**−**	**−**	**−**	**−**	**−**	**−**	**−**
***18***		3-Hydroxyanthranilic acid	**+**	**+**	**+**	**+**	**+**	**+**	**+**	**−**	**+**	**+**	**+**
***19***		4-Aminosalicylic acid	**+**	**+**	**+**	**−**	**−**	**−**	**−**	**−**	**−**	**−**	**−**
***20***		2,6-Dihydroxybenzoic acid	**−**	**−**	**−**	**−**	**−**	**−**	**−**	**−**	**−**	**−**	**−**
***21***		Sodium salicylate	**−**	**−**	**−**	**−**	**−**	**−**	**−**	**−**	**−**	**−**	**−**
***22***		*p*-Hydroxyphenylpyruvic acid	**+**	**+**	**−**	**−**	**−**	**+**	**+**	**−**	**−**	**−**	**−**
***23***		L-DOPA	**−**	**−**	**−**	**−**	**−**	**−**	**−**	**−**	**−**	**−**	**−**
***24***	**Aromatic alcohols**	4-Hydroxybenzyl alcohol	**+**	**+**	**−**	**−**	**−**	**−**	**−**	**−**	**−**	**−**	**−**
***25***		Vanillyl alcohol	**+**	**+**	**+**	**+/−**	**−**	**+**	**+**	**−**	**−**	**−**	**−**
***26***		Isovanillyl alcohol	**+**	**+**	**+**	**−**	**−**	**+**	**+**	**−**	**−**	**+/−**	**−**
***27***		2,3-Dimethoxybenzyl alcohol	**−**	**−**	**−**	**−**	**−**	**−**	**−**	**−**	**−**	**+/−**	**−**
***28***		2,4-Dimethoxybenzyl alcohol	**−**	**−**	**−**	**−**	**−**	**−**	**−**	**−**	**−**	**+/−**	**−**
***29***		2,5-Dimethoxybenzyl alcohol	**−**	**−**	**−**	**−**	**−**	**−**	**−**	**−**	**−**	**+/−**	**−**
***30***		Veratryl alcohol	**−**	**+/−**	**+/−**	**−**	**−**	**−**	**−**	**−**	**−**	**+/−**	**−**
***31***		3,5-Dimethoxybenzyl alcohol	**−**	**+/−**	**+/−**	**−**	**−**	**−**	**−**	**−**	**−**	**+/−**	**−**
***32***		Coniferyl alcohol	**+**	**+**	**+**	**−**	**−**	**+**	**+**	**−**	**−**	**−**	**−**
***33***		Tyrosol	**+**	**+**	**−**	**−**	**−**	**+**	**+**	**+/−**	**−**	**+/−**	**−**
***34***		Phenol	**+**	**+**	**−**	**−**	**−**	**−**	**−**	**−**	**−**	**−**	**−**
***35***		*p*-Cresol	**+**	**+**	**−**	**−**	**−**	**+/−**	**+/−**	**−**	**−**	**−**	**−**
***36***		2,6-Dimethylphenol	**+**	**+**	**+**	**+**	**−**	**+**	**+**	**+**	**−**	**−**	**−**
***37***		Catechol	**+**	**+**	**+**	**+**	**−**	**+**	**+**	**−**	**+**	**+**	**+**
***38***		4-Methylcatechol	**+**	**+**	**+**	**+**	**−**	**+**	**+**	**−**	**+**	**+**	**+**
***39***		Pyrogallol	**+**	**+**	**+**	**+**	**−**	**+**	**+**	**+**	**+**	**+**	**+**
***40***		Isoeugenol	**+**	**+**	**+**	**−**	**−**	**−**	**−**	**−**	**−**	**−**	**−**
***41***		3,4,5-Trimethoxyphenol	**+**	**+**	**+**	**+**	**−**	**+/−**	**+/−**	**−**	**−**	**−**	**−**
***42***		Guaiacol	**+**	**+**	**+**	**+**	**−**	**+**	**+**	**−**	**−**	**−**	**−**
***43***		Hydroquinone	**+**	**+**	**+**	**+**	**−**	**+**	**+**	**+**	**−**	**+**	**+**
***44***		Mesitol	**+**	**+**	**−**	**−**	**−**	**+**	**+**	**−**	**−**	**+/−**	**−**
***45***		3-Methylcatechol	**+**	**+**	**+**	**+**	**−**	**+**	**+**	**+/−**	**+**	**+**	**+**
***46***		Eugenol	**+**	**+**	**+**	**+**	**−**	**+**	**+**	**−**	**−**	**−**	**−**
***47***		Arbutin	**+**	**+**	**+/−**	**−**	**−**	**+**	**+**	**−**	**−**	**−**	**−**
***48***		Resveratrol	**+**	**+**	**+**	**+**	**−**	**+**	**+**	**+**	**+**	**+**	**+**
***49***		Quercetin hydrate	**+**	**+**	**+**	**+**	**+/−**	**+**	**+**	**−**	**+**	**+**	**+**
***50***	**Aromatic ketons**	Acetovanillone	**+**	**−**	**−**	**−**	**−**	**+**	**+**	**−**	**−**	**−**	**−**
***51***		Acetosyringone	**+**	**−**	**−**	**+/−**	**−**	**+**	**+**	**−**	**−**	**−**	**−**
***52***	**Aromatic aldehydes**	*o*-Vanillin	**+**	**+**	**+**	**−**	**−**	**+**	**+**	**−**	**−**	**−**	**−**
***53***		Syringaldehyde	**+**	**+**	**+**	**−**	**−**	**+**	**+**	**−**	**−**	**−**	**−**
***54***		Ethyl vanillin	**+**	**−**	**−**	**−**	**−**	**+**	**+**	**−**	**−**	**−**	**−**
***55***		Vanillin	**+**	**−**	**−**	**−**	**−**	**+/−**	**+/−**	**−**	**−**	**−**	**−**
***56***		Sinapaldehyde	**+**	**+**	**+**	**+**	**−**	**+**	**+**	**+**	**+**	**+**	**+**
***57***		Coniferyl aldehyde	**+**	**+**	**+**	**+/−**	**−**	**+**	**+**	**−**	**+/−**	**+**	**−**
***58***	**Aromatic amines**	Aniline	**+**	**−**	**−**	**−**	**−**	**−**	**−**	**−**	**−**	**−**	**−**
***59***		Tyramine hydrochloride	**+**	**+**	**−**	**−**	**−**	**−**	**−**	**−**	**−**	**+/−**	**−**
***60***		Dopamine hydrochloride	**+**	**+**	**+**	**+**	**−**	**+**	**+**	**+**	**+**	**+**	**+**
***61***	**Aromatic esters**	Methyl vanillate	**+**	**−**	**−**	**−**	**−**	**+**	**+**	**−**	**−**	**−**	**−**
***62***		Methylsyringate	**+**	**+**	**−**	**+/−**	**−**	**+**	**+**	**−**	**−**	**−**	**−**
***63***	**Aromatic amides**	Syringamide	**+**	**+**	**+**	**+**	**−**	**+**	**+**	**−**	**−**	**−**	**−**
***64***		*N*-Hydroxyacetanilide	**+**	**+**	**+/−**	**−**	**−**	**+**	**+**	**−**	**−**	**−**	**−**
***65***	**Polyphenol**	Tannic acid	**+**	**+**	**+**	**+/−**	**−**	**+**	**+**	**−**	**+**	**+**	**+**
***66***	***N*** **-heterocycles**	HOBt	**−**	**−**	**−**	**−**	**−**	**−**	**−**	**−**	**−**	**−**	**−**
***67***		*N*-Hydroxyphthalimide	**+**	**+**	**+**	**+/−**	**−**	**−**	**−**	**−**	**+**	**+**	**+**
***68***		HOAt	**−**	**−**	**−**	**−**	**−**	**−**	**−**	**−**	**−**	**−**	**−**
***69***		DHBT	**−**	**−**	**−**	**−**	**−**	**−**	**−**	**−**	**−**	**−**	**−**
***70***		Violuric acid hydrate	**−**	**−**	**−**	**−**	**−**	**−**	**−**	**−**	**−**	**−**	**−**
***71***		TEMPO	**−**	**−**	**−**	**−**	**−**	**−**	**−**	**−**	**−**	**−**	**−**
***72***		TEMPOL	**−**	**−**	**−**	**−**	**−**	**−**	**−**	**−**	**−**	**−**	**−**
***73***		3-Carbamoyl-PROXYL	**−**	**−**	**−**	**−**	**−**	**−**	**−**	**−**	**−**	**−**	**−**
***74***		1-(3-Sulfophenyl)-3-methyl-2-pyrazolin-5-one	**+**	**+**	**+**	**+**	**−**	**+**	**+**	**−**	**−**	**−**	**−**
***75***		1-(4-Sulfophenyl)-3-methyl-5-pyrazolone	**+**	**+**	**+**	**+**	**−**	**+**	**+**	**−**	**−**	**−**	**−**
***76***		Methyl viologen dichlorid hydrate	**−**	**−**	**−**	**−**	**−**	**−**	**−**	**−**	**−**	**−**	**−**
***77***	**Aromatic azo compounds**	ABTS	**+**	**+**	**+**	**+**	**+/−**	**+**	**+**	**+**	**+**	**+**	**−**
***78***		Syringaldazine	**+**	**−**	**+**	**+**	**+/−**	**+**	**+**	**−**	**+**	**+**	**+**
***79***	**Triphenyl compounds**	Phenolphtalein	**+**	**+**	**+**	**−**	**−**	**−**	**−**	**+**	**−**	**+**	**+**
***80***		Triphenylamine	**+**	**+**	**+**	**+**	**−**	**−**	**+**	**−**	**+**	**+**	**+/−**
***81***		Phenol red	**+**	**−**	**−**	**−**	**−**	**−**	**−**	**−**	**−**	**−**	**−**
***82***		Cresol red sodium salt	**+**	**+**	**+**	**−**	**−**	**+**	**+**	**−**	**−**	**+/−**	**−**
***83***	**Chroman**	(+)-Catechin hydrate	**+**	**+**	**+**	**+**	**−**	**+**	**+**	**−**	**+**	**+**	**+**
***84***		(**−**)-Epicatechin	**+**	**+**	**+**	**+**	**−**	**+**	**+**	**−**	**+**	**+**	**+**
***85***	**Phenothiazines**	Phenothiazine	**+**	**+**	**−**	**+/−**	**−**	**+**	**+**	**+**	**+**	**+**	**+**
***86***		Promazine hydrochloride	**+/−**	**−**	**−**	**−**	**−**	**+**	**+**	**−**	**+**	**+**	**−**
***87***	**Benzonitriles**	2,3-Dimethoxybenzonitrile	**−**	**−**	**−**	**−**	**−**	**+/−**	**+/−**	**−**	**−**	**−**	**−**
***88***		3,5-Dimethoxybenzonitrile	**−**	**−**	**−**	**−**	**−**	**−**	**−**	**−**	**−**	**−**	**−**
***89***	**Naphthalenes**	1-Nitroso-2-naphthol-3,6-disulfonic acid	**+**	**−**	**−**	**−**	**−**	**+**	**+**	**−**	**−**	**−**	**−**
***90***		2-Nitroso-1-naphthol-4-sulfonic acid	**+**	**+**	**−**	**−**	**−**	**+**	**+**	**−**	**−**	**−**	**−**
***91***		1-Amino-2-naphthol-4-sulfonic acid	**+**	**−**	**−**	**−**	**−**	**+**	**−**	**−**	**−**	**−**	**−**

Substrates are categorized into 15 groups according to chemical structures. Activity towards a substrate based on a change of absorbance is given as (+), no activity as (−) and ambiguous activity (+/−) when not clear. Abbreviation: b - bacterial f - fungal, and p - plant. Tve: *T. versicolor*, Abi: *A. bisporus*, Mth: *M. thermophila*, Rve: *R. vernificera*, Cur: *Cucurbita* (AsOX), Bsu: *B. subtilis*, Bpu: *B. pumilus*, Spr: *S. pristinaespiralis*, Gfo: *G. forsetii*, Mtr: *M. tractuosa* and Sli: *S. linguale*.

### Substrate screen

Substrate spectra of numerous laccases and LMCOs from bacterial and fungal origin have been reported. However, comparison of the data is difficult due to the choice of compound and experimental procedure [8], [29], [32], [33], [34]. In an attempt to reveal the substrate spectrum of the selected enzymes, fungal, bacterial and plant laccases and LMCOs were subjected to a simple substrate screen (Table 3). In total, 91 compounds were screened, comprising potential substrates of synthetic and natural origin. The compounds were grouped in 15 clusters which were: aromatic carboxylic acids, aromatic alcohols, aromatic ketons, aromatic aldehydes, aromatic amines, aromatic esters, aromatic amides, polyphenols, *N*-heterocycles, aromatic azo compounds, triphenyl compounds, chromans, phenothiazines, benzonitriles and naphtalenes. The activity was measured at a substrate concentration of 1 mM at pH 6. A UV-Vis scan between 230–700 nm was recorded prior to enzyme addition and after 24 h reaction time. By this method only substrates leading to a changed UV spectrum upon oxidation were detectable. The enzyme substrate combinations which resulted in a clearly distinct changed UV spectrum were marked as (+), whereas unchanged spectra were marked as (−); in a number of cases the classification between (+) and (−) was not clear, and these were marked as (+/−). Control reactions lacking enzyme were performed in parallel to exclude the possibility of non-enzymatic oxidation. To subtract any contaminating activity originating from other than the respective LMCO, samples from *E. coli* cells harboring the empty vector and heat inactivated commercial laccase and LMCOs were also assayed, resulting in a non-specific signal that was independent of LMCOs.

**Figure 2:**
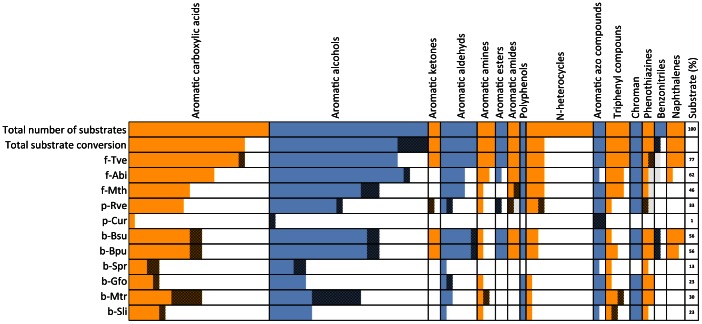
Activity pattern of LMCOs and laccase. Each colored bar gives the total number of converted substrates within a substrate cluster (15 in total). Solid color indicates activity (+) and shaded bars indicated activity where the UV-Vis signal was not conclusive (+/−). The top bar gives the total number of substrate compounds in each cluster, whereas the number of oxidized substrates by at least one of the LMCOs or laccase is given in the second row (total substrate conversion). The percentage of oxidized substrate from the total 91 compounds by the individual enzyme is given in the last column.

Table 3 summarizes the substrate screen in detail. Each of the 91 chemical structures is mentioned by name in Table 3 and has been depicted in Table S1 in the supplementary section. LMCO from *Trametes versicolor* (f-Tve) was found to show the broadest substrate spectrum. Activity towards 70 compounds was found. For two substrates (**15**, **86**) the UV-signal changed only slightly compared to the control and was therefore given with reservations given as (+/−). Figure 2 shows a condensed overview of the substrate screen. The activity pattern for the 15 substrate clusters is represented schematically. Unambiguous, unique activity was found for substrates **2**, **7**, **13**, **17**, **55**, **58** and **81**, not taking (+/−) ranked entries into account. *A. bisporus* LMCO (f-Abi) was active towards 56 compounds and *M. thermophila* (f-Mth) LMCO towards 42 compounds, all of which were also oxidized by *T. versicolor*. Substrates **14**, **19**, **24**, **34**, **35**, **40** and **59** were only oxidized by fungal LMCOs, if neglecting activity for bacterial LMCOs which were ranked as (+/−). A striking difference to all other LMCOs was seen for the plant enzyme from *Cucurbita* (p-Cur): it showed clear oxidation only of substrate **18**, the phenolic 3-hydroxyanthranilic acid. Even classical laccase substrates such as ABTS (**77**) or syringaldazine (**78**) were not clearly identified (+/−) and it is interesting that a particular aromatic compound is a substrate of this enzyme and makes it a “laccase-like” enzyme to some extent. In fact, looking at the sequence conservation in the active site, *i.e.* the copper binding motives, this enzyme shares all relevant residues with the other LMCOs. However, this enzyme has been classified to the E.C. class 1.10.3.3. of ascorbate oxidases that is distinct from the E.C. 1.10.3.2. class of laccases. This example demonstrates that the E.C. classification has only limited value and involves both overlap and ambiguity for certain enzymes.

The enzyme from *R. vernificera* (p-Rve) was active towards 30 compounds, all of which were also oxidized by the fungal LMCOs (**78** not by f-Abi) as well as the two *Bacillus* LMCOs (**41** with reservations).The latter two enzymes performed identically, with the exception of substrates **80** and **91**, which were only oxidized by *B. pumilus* and *B. subtilis* LMCOs, respectively. Clear activity was found towards 51 substrates. Again, for 6 substrates (**3**, **15**, **35**, **41**, **55** and **87**) detection of activity by this method can only be given with reservations. The three well-characterized *B. subtilis*, *B. pumilus* and *B. licheniformis* LMCOs exhibit 61–67% amino acid sequence identity. In agreement with the observed similarity in the substrate range for b-Bpu and b-Bsu, the reported *k*_cat_ and *K*_m_ values for the three standard laccase substrates ABTS, DMP and syringaldazine are all in the same order of magnitude [11], [23], [29]. For the four previously undescribed bacterial LMCOs, a reduced diversity in terms of substrate range was found. The most versatile enzyme was that from *M. tractuosa* with activity towards 27 compounds, followed by the *G. forsetii* and *S. linguale* enzymes with 21 positive compounds, all of which were substrates for the other LMCOs. The *S. pristinaespiralis* enzyme, which has a considerably lower molecular weight than the other LCMOs (Tab. 1), exhibited the most limited substrate range, with only 12 oxidized compounds. All of these compounds were also oxidized by the other LMCOs. The reduced number of phenolic compounds oxidized by the LMCOs of Gram-negative bacteria observed in this study may be connected with a different type of natural function. The periplasmic LMCO CueO from *E. coli* was suggested to be mainly involved in cuprous oxide detoxification and copper homeostatis [35]. Similarly to the presumably periplasmic b-MtL, b-Gfo and b-Sli LMCOs, CueO oxidizes ABTS but not DMP or syringaldehyde in the absence of high concentrations of copper [36]. It was hypothesized that methionine-rich α-helixes of CueO hinder the access of bulky organic substrates and confer specificity for cuprous oxide [36]. The primary structures of b-MtL, b-Gfo and b-Sli also contain such methionine rich regions (Fig. 1 B). Thus, they may rather be cuprous oxide oxidases instead of enzymes with a laccase-like substrate preference, in particular with respect to methoxy-substituted phenols.

As discussed previously [11] most of the oxidized substrates were substituted phenols with at least one *ortho* or *para* – substituent adjacent to the phenoxy-OH, bearing a lone pair of electrons and thus contributing to the positive mesomeric effect, e.g. substrates **4**, **6**, **12** and **56**. Electron abstraction is facilitated due to the increased electron density at the phenoxy-OH, resulting in a phenoxy radical. Substrates **4**, **6**, **12**, **39**, **45**, **56**, **60**, **85**, and **48** were substrates for all tested LMCOs, apart from *Cucurbita* (AsOX), if including the combinations where we have reservations. Activity towards the latter substrate cannot be explained based on the phenol substitution pattern. The same holds true for substrates such as **2**, **3**, **7**, **13**, **19**, **22**, **24**, **33**, **34**, **35**, **36**, **41**, **43**, **44**, **47**, **59**, **64**, **79**, **81** and **82**. Substrates, other than **67**, **74** and **75** from the N-heterocylic group are not applicable for the screening with the described methodology as they and their oxidation products are not UV visible. Nevertheless, they should be considered as substrates and even mediators [37].

The substitution pattern of phenols is linked to the activity and it was found that *ortho*- substituted phenols were better substrates than *para*-substituted compounds and *meta*-substituted compounds showed the lowest reaction rate [8], [38]. Whether a compound is oxidized by a laccase or LMCOs and at which rate, can also be attributed to the difference in the redox potential or ionization energy [34], [39]. However, within this study we focused on the elucidation of the substrates spectrum regardless the physico-chemical properties solely by UV-Vis spectrophometry.

## Conclusion

From the substrate screen it can be concluded that the chosen LMCOs are capable of oxidizing a chemically diverse group of substrates. Notably, the fungal LMCOs from *T. versicolor* and *A. bisporus* and both bacterial enzymes from *Bacillus* oxidize 77, 62 and 56% of the total number of substrates, respectively. A poor substrate range was found for b-Spr, b-Gfo, b-Mtr, b-Sli LMCOs, all of bacterial origin and perhaps representing a separate subclass within the LMCOs. The plant ascorbate oxidase from *Cucurbita* oxidized only one substrate, although all the copper binding residues are present and identical. Our example emphasizes the issue of how to identify and classify laccases from amino acid structure sequences. This becomes even more important in view of the fact that a vast amount of so far experimentally uncharacterized sequences have been annotated as laccases, mostly based on the presence of the conserved copper ligand motifs. Sequences were obtained by database mining and phylogenetic analysis and are characterized by a high diversity [13], [14]. The biological function of the characterized enzymes is also assumed to be diverse, although their natural substrates(s) are mostly unknown. As discussed previously, the classification of laccases or LMCOs by substrate alone is inadequate, due to their highly diverse and often overlapping substrate spectra. Focusing solely on the presence of the coordinating amino acid residues for the copper centers is also not sufficient. Clearly, residues other than the copper coordinating ones should also be considered. The ‘holy grail’ in laccase research may lie in the identification of reliable structural features as well as in an adequate definition of their substrates.

Besides the need for a clear and consistent classification, these enzymes have great biotechnological potential. Fungal LMCOs have the broadest substrate range; however, they are notoriously difficult to overexpress in heterologous hosts, and potentially required enzyme engineering is time consuming. LMCOs from Gram-negative bacteria and *Streptomyces* seem to suffer from a reduced substrate range, and bacterial laccases suffer in general from a fairly low production yield in bacterial expression systems. Once the obstacles regarding the yields of active protein in *E. coli* have been removed, LMCOs from *Bacillus* species [11], [22], [29] seem to have the best potential for industrial application, especially with respect to their broad substrate and mediator range and the available molecular biological tools, which would allow to improve the enzyme further, for example by directed evolution.

## Supporting Information

Table S1 **Substrate screen for the 11 studied laccase and LMCOs against the 91 tested substrates.**
(DOCX)
