# Supplementary material for: Laccase versus Laccase-Like Multi-Copper Oxidase: A Comparative Study of Similar Enzymes with Diverse Substrate Spectra
Source: PLoS One. 2013 Jun 3;8(6):e65633. doi: 10.1371/journal.pone.0065633 (PMC3670849; doi:10.1371/journal.pone.0065633)
Supplement: Table S1 — Substrate screen for the 11 studied laccase and LMCOs against the 91 tested substrates. (DOCX) [file pone.0065633.s001.docx]

**S1: Supplementary table**.

| ***No*** |  | **Compound** |  | f-Tve | f-Abi | f-Mth | p-Rve | p-Cur | b-Bsu | b-Bpu | b-Spr | b-Gfo | b-Mtr | b-Sli |
| --- | --- | --- | --- | --- | --- | --- | --- | --- | --- | --- | --- | --- | --- | --- |
| ***1*** | **Aromatic carboxylic acids** | *Trans*-cinnamic acid[ |  | **—** | **—** | **—** | **—** | **—** | **—** | **—** | **—** | **—** | **—** | **—** |
| ***2*** |  | *m*-Coumaric acid |  | **+** | **—** | **—** | **—** | **—** | **—** | **—** | **—** | **—** | **—** | **—** |
| ***3*** |  | *p*-Coumaric acid |  | **+** | **+** | **—** | **—** | **—** | **+/—** | **+/—** | **—** | **—** | **—** | **—** |
| ***4*** |  | Caffeic acid |  | **+** | **+** | **+** | **+** | **—** | **+** | **+** | **+/—** | **+** | **+** | **+** |
| ***5*** |  | Ferulic acid |  | **+** | **+** | **+** | **+** | **—** | **+** | **+** | **+/—** | **—** | **+/—** | **+/—** |
| ***6*** |  | Sinapic acid |  | **+** | **+** | **+** | **+** | **—** | **+** | **+** | **+** | **+/—** | **+** | **+** |
| ***7*** |  | 4-Hydroxybenzoic acid |  | **+** | **—** | **—** | **—** | **—** | **—** | **—** | **—** | **—** | **—** | **—** |
| ***8*** |  | 3,4 Dihydroxybenzoic acid |  | **+** | **+** | **+** | **+** | **—** | **+** | **+** | **—** | **—** | **+/—** | **—** |
| ***9*** |  | Gallic acid |  | **+** | **+** | **+** | **+** | **—** | **+** | **+** | **—** | **+** | **+** | **—** |
| ***10*** |  | Syringic acid |  | **+** | **+** | **+** | **+** | **—** | **+** | **+** | **+** | **—** | **+** | **+** |
| ***11*** |  | 3-Amino-4 hydroxybenzoic acid |  | **+** | **+** | **+** | **+** | **—** | **+** | **+** | **—** | **—** | **—** | **—** |
| ***12*** |  | 4-Amino-3 hydroxybenzoic acid |  | **+** | **+** | **+** | **+** | **—** | **+** | **+** | **+** | **+** | **+** | **+** |
| ***13*** |  | 3-Fluoro-4-hydroxybenzoic acid |  | **+** | **—** | **—** | **—** | **—** | **—** | **—** | **—** | **—** | **+/—** | **—** |
| ***14*** |  | 4-Dimethylaminobenzoic acid |  | **+** | **+** | **—** | **—** | **—** | **—** | **—** | **—** | **—** | **+/—** | **—** |
| ***15*** |  | Vanillic acid |  | **+/—** | **+** | **—** | **—** | **—** | **+/—** | **+/-** | **—** | **—** | **+/—** | **—** |
| ***16*** |  | Anthranilic acid |  | **+** | **—** | **—** | **—** | **—** | **—** | **—** | **—** | **—** | **+** | **—** |
| ***17*** |  | 3-Dimethylamino benzoic acid |  | **+** | **—** | **—** | **—** | **—** | **—** | **—** | **—** | **—** | **—** | **—** |
| ***18*** |  | 3-Hydroxyanthranilic acid |  | **+** | **+** | **+** | **+** | **+** | **+** | **+** | **—** | **+** | **+** | **+** |
| ***19*** |  | 4-Aminosalicylic acid |  | **+** | **+** | **+** | **—** | **—** | **—** | **—** | **—** | **—** | **—** | **—** |
| ***20*** |  | 2,6 Dihydroxybenzoic acid |  | **—** | **—** | **—** | **—** | **—** | **—** | **—** | **—** | **—** | **—** | **—** |
| ***21*** |  | Sodium salicylate |  | **—** | **—** | **—** | **—** | **—** | **—** | **—** | **—** | **—** | **—** | **—** |
| ***22*** |  | *p*-Hydroxyphenylpyruvic acid |  | **+** | **+** | **—** | **—** | **—** | **+** | **+** | **—** | **—** | **—** | **—** |
| ***23*** |  | L-DOPA |  | **—** | **—** | **—** | **—** | **—** | **—** | **—** | **—** | **—** | **—** | **—** |
| ***24*** | **Aromatic alcohols** | 4-Hydroxybenzyl alcohol |  | **+** | **+** | **—** | **—** | **—** | **—** | **—** | **—** | **—** | **—** | **—** |
| ***25*** |  | Vanillyl alcohol |  | **+** | **+** | **+** | **+/—** | **—** | **+** | **+** | **—** | **—** | **—** | **—** |
| ***26*** |  | Isovanillyl alcohol |  | **+** | **+** | **+** | **—** | **—** | **+** | **+** | **—** | **—** | **+/—** | **—** |
| ***27*** |  | 2,3-Dimethoxybenzyl alcohol |  | **—** | **—** | **—** | **—** | **—** | **—** | **—** | **—** | **—** | **+/—** | **—** |
| ***28*** |  | 2,4-Dimethoxybenzyl alcohol |  | **—** | **—** | **—** | **—** | **—** | **—** | **—** | **—** | **—** | **+/—** | **—** |
| ***29*** |  | 2,5-Dimethoxybenzyl alcohol |  | **—** | **—** | **—** | **—** | **—** | **—** | **—** | **—** | **—** | **+/—** | **—** |
| ***30*** |  | Veratryl alcohol |  | **—** | **+/—** | **+/—** | **—** | **—** | **—** | **—** | **—** | **—** | **+/—** | **—** |
| ***31*** |  | 3,5-Dimethoxybenzyl alcohol |  | **—** | **+/—** | **+/—** | **—** | **—** | **—** | **—** | **—** | **—** | **+/—** | **—** |
| ***32*** |  | Coniferyl alcohol |  | **+** | **+** | **+** | **—** | **—** | **+** | **+** | **—** | **—** | **—** | **—** |
| ***33*** |  | Tyrosol |  | **+** | **+** | **—** | **—** | **—** | **+** | **+** | **+/—** | **—** | **+/—** | **—** |
| ***34*** |  | Phenol |  | **+** | **+** | **—** | **—** | **—** | **—** | **—** | **—** | **—** | **—** | **—** |
| ***35*** |  | *p*-Cresol |  | **+** | **+** | **—** | **—** | **—** | **+/—** | **+/—** | **—** | **—** | **—** | **—** |
| ***36*** |  | 2,6-Dimethylphenol |  | **+** | **+** | **+** | **+** | **—** | **+** | **+** | **+** | **—** | **—** | **—** |
| ***37*** |  | Catechol |  | **+** | **+** | **+** | **+** | **—** | **+** | **+** | **—** | **+** | **+** | **+** |
| ***38*** |  | 4-Methylcatechol |  | **+** | **+** | **+** | **+** | **—** | **+** | **+** | **—** | **+** | **+** | **+** |
| ***39*** |  | Pyrogallol |  | **+** | **+** | **+** | **+** | **—** | **+** | **+** | **+** | **+** | **+** | **+** |
| ***40*** |  | Isoeugenol |  | **+** | **+** | **+** | **—** | **—** | **—** | **—** | **—** | **—** | **—** | **—** |
| ***41*** |  | 3,4,5-Trimethoxyphenol |  | **+** | **+** | **+** | **+** | **—** | **+/—** | **+/—** | **—** | **—** | **—** | **—** |
| ***42*** |  | Guaiacol |  | **+** | **+** | **+** | **+** | **—** | **+** | **+** | **—** | **—** | **—** | **—** |
| ***43*** |  | Hydroquinone |  | **+** | **+** | **+** | **+** | **—** | **+** | **+** | **+** | **—** | **+** | **+** |
| ***44*** |  | Mesitol |  | **+** | **+** | **—** | **—** | **—** | **+** | **+** | **—** | **—** | **+/—** | **—** |
| ***45*** |  | 3-Methylcatechol |  | **+** | **+** | **+** | **+** | **—** | **+** | **+** | **+/—** | **+** | **+** | **+** |
| ***46*** |  | Eugenol |  | **+** | **+** | **+** | **+** | **—** | **+** | **+** | **—** | **—** | **—** | **—** |
| ***47*** |  | Arbutin |  | **+** | **+** | **+/—** | **—** | **—** | **+** | **+** | **—** | **—** | **—** | **—** |
| ***48*** |  | Resveratrol |  | **+** | **+** | **+** | **+** | **—** | **+** | **+** | **+** | **+** | **+** | **+** |
| ***49*** |  | Quercetin hydrate |  | **+** | **+** | **+** | **+** | **+/—** | **+** | **+** | **—** | **+** | **+** | **+** |
| ***50*** | **Aromatic ketons** | Acetovanillone |  | **+** | **—** | **—** | **—** | **—** | **+** | **+** | **—** | **—** | **—** | **—** |
| ***51*** |  | Acetosyringone |  | **+** | **—** | **—** | **+/—** | **—** | **+** | **+** | **—** | **—** | **—** | **—** |
| ***52*** | **Aromatic aldehyds** | *o*-Vanillin |  | **+** | **+** | **+** | **—** | **—** | **+** | **+** | **—** | **—** | **—** | **—** |
| ***53*** |  | Syringaldehyde |  | **+** | **+** | **+** | **—** | **—** | **+** | **+** | **—** | **—** | **—** | **—** |
| ***54*** |  | Ethyl vanillin |  | **+** | **—** | **—** | **—** | **—** | **+** | **+** | **—** | **—** | **—** | **—** |
| ***55*** |  | Vanillin |  | **+** | **—** | **—** | **—** | **—** | **+/—** | **+/—** | **—** | **—** | **—** | **—** |
| ***56*** |  | Sinapaldehyde |  | **+** | **+** | **+** | **+** | **—** | **+** | **+** | **+** | **+** | **+** | **+** |
| ***57*** |  | Coniferyl aldehyde |  | **+** | **+** | **+** | **+/—** | **—** | **+** | **+** | **—** | **+/—** | **+** | **—** |
| ***58*** | **Aromatic amines** | Aniline |  | **+** | **—** | **—** | **—** | **—** | **—** | **—** | **—** | **—** | **—** | **—** |
| ***59*** |  | Tyramine hydrochloride |  | **+** | **+** | **—** | **—** | **—** | **—** | **—** | **—** | **—** | **+/—** | **—** |
| ***60*** |  | Dopamine hydrochloride |  | **+** | **+** | **+** | **+** | **—** | **+** | **+** | **+** | **+** | **+** | **+** |
| ***61*** | **Aromatic esters** | Methyl vanillate |  | **+** | **—** | **—** | **—** | **—** | **+** | **+** | **—** | **—** | **—** | **—** |
| ***62*** |  | Methylsyringate |  | **+** | **+** | **—** | **+/—** | **—** | **+** | **+** | **—** | **—** | **—** | **—** |
| ***63*** | **Aromatic amides** | Syringamide |  | **+** | **+** | **+** | **+** | **—** | **+** | **+** | **—** | **—** | **—** | **—** |
| ***64*** |  | *N*-Hydroxyacetanilide |  | **+** | **+** | **+/—** | **—** | **—** | **+** | **+** | **—** | **—** | **—** | **—** |
| ***65*** | **Polyphenol** | Tannic acid |  | **+** | **+** | **+** | **+/—** | **—** | **+** | **+** | **—** | **+** | **+** | **+** |
| ***66*** | **N-heterocycles** | HOBt |  | **—** | **—** | **—** | **—** | **—** | **—** | **—** | **—** | **—** | **—** | **—** |
| ***67*** |  | *N*-Hydroxyphthalimide |  | **+** | **+** | **+** | **+/—** | **—** | **—** | **—** | **—** | **+** | **+** | **+** |
| ***68*** |  | HOAt |  | **—** | **—** | **—** | **—** | **—** | **—** | **—** | **—** | **—** | **—** | **—** |
| ***69*** |  | DHBT |  | **—** | **—** | **—** | **—** | **—** | **—** | **—** | **—** | **—** | **—** | **—** |
| ***70*** |  | Violuric acid hydrate |  | **—** | **—** | **—** | **—** | **—** | **—** | **—** | **—** | **—** | **—** | **—** |
| ***71*** |  | TEMPO |  | **—** | **—** | **—** | **—** | **—** | **—** | **—** | **—** | **—** | **—** | **—** |
| ***72*** |  | TEMPOL |  | **—** | **—** | **—** | **—** | **—** | **—** | **—** | **—** | **—** | **—** | **—** |
| ***73*** |  | 3-Carbamoyl-PROXYL |  | **—** | **—** | **—** | **—** | **—** | **—** | **—** | **—** | **—** | **—** | **—** |
| ***74*** |  | 1-(3-Sulfophenyl)-3-methyl-2-pyrazolin-5-one |  | **+** | **+** | **+** | **+** | **—** | **+** | **+** | **—** | **—** | **—** | **—** |
| ***75*** |  | 1-(4-Sulfophenyl)-3-methyl-5-pyrazolone |  | **+** | **+** | **+** | **+** | **—** | **+** | **+** | **—** | **—** | **—** | **—** |
| ***76*** |  | Methyl viologen dichlorid hydrate |  | **—** | **—** | **—** | **—** | **—** | **—** | **—** | **—** | **—** | **—** | **—** |
| ***77*** | **Aromatic azo compounds** | ABTS |  | **+** | **+** | **+** | **+** | **+/—** | **+** | **+** | **+** | **+** | **+** | **—** |
| ***78*** |  | Syringaldazine |  | **+** | **—** | **+** | **+** | **+/—** | **+** | **+** | **—** | **+** | **+** | **+** |
| ***79*** | **Triphenyl compounds** | Phenolphtalein |  | **+** | **+** | **+** | **—** | **—** | **—** | **—** | **+** | **—** | **+** | **+** |
| ***80*** |  | Triphenylamine |  | **+** | **+** | **+** | **+** | **—** | **—** | **+** | **—** | **+** | **+** | **+/—** |
| ***81*** |  | Phenol red |  | **+** | **—** | **—** | **—** | **—** | **—** | **—** | **—** | **—** | **—** | **—** |
| ***82*** |  | Cresol red sodium salt |  | **+** | **+** | **+** | **—** | **—** | **+** | **+** | **—** | **—** | **+/—** | **—** |
| ***83*** | **Chroman** | (+)-Catechin hydrate |  | **+** | **+** | **+** | **+** | **—** | **+** | **+** | **—** | **+** | **+** | **+** |
| ***84*** |  | (-)-Epicatechin |  | **+** | **+** | **+** | **+** | **—** | **+** | **+** | **—** | **+** | **+** | **+** |
| ***85*** | **Phenothiazines** | Phenothiazine |  | **+** | **+** | **—** | **+/—** | **—** | **+** | **+** | **+** | **+** | **+** | **+** |
| ***86*** |  | Promazine hydrochloride |  | **+/—** | **—** | **—** | **—** | **—** | **+** | **+** | **—** | **+** | **+** | **—** |
| ***87*** | **Benzonitriles** | 2,3-Dimethoxybenzonitrile |  | **—** | **—** | **—** | **—** | **—** | **+/—** | **+/—** | **—** | **—** | **—** | **—** |
| ***88*** |  | 3,5-Dimethoxybenzonitrile |  | **—** | **—** | **—** | **—** | **—** | **—** | **—** | **—** | **—** | **—** | **—** |
| ***89*** | **Naphthalenes** | 1-Nitroso-2-naphthol-3,6-disulfonic acid |  | **+** | **—** | **—** | **—** | **—** | **+** | **+** | **—** | **—** | **—** | **—** |
| ***90*** |  | 2-Nitroso-1-naphthol-4-sulfonic acid |  | **+** | **+** | **—** | **—** | **—** | **+** | **+** | **—** | **—** | **—** | **—** |
| ***91*** |  | 1-Amino-2-naphthol-4-sulfonic acid |  | **+** | **—** | **—** | **—** | **—** | **+** | **—** | **—** | **—** | **—** | **—** |

Substrates are categorized into 15 groups according to chemical sturctures, which are shown. Activity towards a substrate based on a change of absorbance is given as (+), no activity as (-) and (+/-) when ambiguous. Abbreviation: f - fungal, p - plant and b - bacterial. Tve: *T. versicolor*, Abi: *A. bisporus*, Mth: *M. thermophile*, Rve: *R. vernificera*, Cur: *Cucurbita* (Asox), Bsu: *B. subtilis*, Bpu: *B. pumilus*, Spr: *S. pristinaespiralis*, Gfo: *G. forsetii*, Mtr: *M. tractuosa* and Sli: *S. linguale*
